# East Indian Sandalwood Oil (EISO) Alleviates Inflammatory and Proliferative Pathologies of Psoriasis

**DOI:** 10.3389/fphar.2017.00125

**Published:** 2017-03-16

**Authors:** Manju Sharma, Corey Levenson, Ian Clements, Paul Castella, Kurt Gebauer, Michael E. Cox

**Affiliations:** ^1^The Vancouver Prostate Centre, VancouverBC, Canada; ^2^Santalis Pharmaceuticals, Inc., San AntonioTX, USA; ^3^Fremantle Dermatology, FremantleWA, Australia

**Keywords:** skin organoid, cytokine, chemokine, sandalwood oil, anti-inflammatory, anti-proliferative, immunomodulation

## Abstract

Psoriasis, a chronic inflammatory skin disease marked by hyper proliferation and aberrant differentiation of keratinocytes, affects 2–3% of the world’s population. Research into the pathogenesis of psoriasis has been hampered by the lack of models that accurately reflect the biology of the psoriatic phenotype. We have previously reported that East Indian Sandalwood oil (EISO) has significant anti-inflammatory properties in skin models and hypothesized that EISO might provide therapeutic benefit to psoriasis patients due to its anti-inflammatory and anti-proliferative properties. Here we present interim results from an on-going proof-of-concept Phase 2 clinical trial in which topically applied EISO is demonstrating to be well tolerated and helpful in alleviating mild to moderate psoriasis symptoms. This led us to evaluate the ability of EISO to affect the psoriatic phenotype using MatTek Corporation reconstituted organotypic psoriatic and normal human skin models. EISO had no impact on the phenotype of the normal skin tissue model, however, EISO treatment of the psoriasis tissue model reverted psoriatic pathology as demonstrated by histologic characterization and expression of keratinocyte proliferation markers, Ki67 and psoriasin. These phenotypic affects correlated with suppressed production of ENA-78, IL-6, IL-8, MCP-1, GM-CSF, and IL-1β. Demonstration of the ability of EISO to abrogate these psoriasis symptoms in well-characterized *in vitro* psoriatic tissue models, supports the hypothesis that the clinically observed symptom alleviation is due to suppression of intrinsic tissue inflammation reactions in afflicted lesions. This study presents a systematic approach to further study the underlying mechanisms that cause psoriasis, and presents data supporting the potential of EISO as a new ethnobotanical therapeutic concept to help direct and accelerate the development of more effective therapies.

## Introduction

Psoriasis is a chronic T-cell-mediated autoimmune inflammatory skin disease marked by hyper-proliferation and aberrant keratinocyte differentiation affecting 2–3% of the world’s population (Gisondi et al., 2011). Psoriasis patients suffer from impaired quality of life, psychosocial problems and emotional distress (Strand et al., 2013; Belge et al., 2014; Mease and Armstrong, 2014). The pathogenesis of psoriasis involves changes in the innate (macrophages, dendritic cells, monocytes, neutrophils, endothelial cells, and keratinocytes) and acquired immune systems (T lymphocytes). The acquired and innate immune systems interact, resulting in the production of growth factors, cytokines and chemokines. Such triggers as injury, infections, stress, and drugs initiate the inflammatory process resulting in keratinocyte hyperproliferation (Papp et al., 2012; Schafer, 2012; Dubois Declercq and Pouliot, 2013; Mease and Armstrong, 2014).

Psoriasis pathogenesis has been described as being caused by cytokine/chemokine-mediated interactions between keratinocytes and endothelial cells and the innate and adaptive immune systems (Heidenreich et al., 2009; Yazdi et al., 2016). It is thought that in susceptible individuals, a variety of insults activate release of proinflammatory cytokines such as tumor necrosis factor-α (TNF-α) and interleukins (ILs) from skin cells. This activates antigen presenting cells in the skin that migrate to regional lymph nodes to activate T cells (Dubois Declercq and Pouliot, 2013; Garcia-Perez et al., 2013). These CD4+, CD8+, and Th17 T cells then return to the skin, where, through the release of interferon (INF)-γ, TNF-α, IL-6, IL-22, perforin, and granzyme B, activate macrophages, neutrophils, dendiritic cells and promote proliferation of keratinocytes.

The pathogenesis of psoriasis involves genetic, environmental, and immunological factors that often require long-term management. Conventional treatments for psoriasis are typically topical therapies, all of which come with potentially severe side effects (Schafer, 2012; Ports et al., 2013; Perez, 2013; Hsu and Armstrong, 2014). Topical therapies available for mild-to-moderate psoriasis include keratolytics (salicylic acid, urea); topical retinoids (Tazarotene); topical vitamin analogs (calcitriol, tacalcitol, and calcipotriol); calcineurin inhibitors (pimecrolimus and tacrolimus); emollients; dithranol, and tars (Laws and Young, 2010; Murphy and Reich, 2011). Although topical corticosteroids remain first-line treatment that help mitigate all grades of psoriasis, adverse effects such as atrophy, striae and/or telangiectases prevent their long-term utilization (Schoepe et al., 2006; Horn et al., 2010; Laws and Young, 2010). The combination of corticosteroids and vitamin D analogs show superior efficacy as compared to monotherapy; however, the side effects like skin irritation, erythema and edema are seen in up to 35% of patients (Menter and Griffiths, 2007). Salicylic acid is safe to use as a treatment for psoriasis but the long-term use of this drug may result in systemic salicylic acid toxicity (Lebwohl, 1999). Tazarotene, a retinoid derivative, is reported to cause skin irritation in up to 30% of users (Weinstein et al., 2003). While, more recently developed biological agents offer improved anti-psoriatic therapeutic responses, they too pose risk of adverse effect, are expensive, and the potential for development of tolerance or resistance can limit their use (Gottlieb et al., 2008). Hence there is a need to develop new therapies (Schafer, 2012; Garcia-Perez et al., 2013).

Natural products (NPs) represent a vast structural diversity of bioactive small molecules. Evolved as efficient host defense agents, NPs remain the best source and inspiration identifying and developing new drugs (Newman and Cragg, 2012). For more than 4,000 years, the East Indian sandalwood tree, *Santulum album* L. (Santalaceae) has been used in religious rituals, as fragrance, flavoring and as an Ayurvedic medicine as management for depression, inflammation reactions, as an insecticide, an anti-fungal, a sedative and an astringent (Burdock and Carabin, 2008; So et al., 2010; Zhang and Dwivedi, 2011; Saraswati et al., 2013; Lee et al., 2015). These antiseptic, anti-inflammatory and anti-proliferative properties make East Indian Sandalwood oil (EISO) an attractive potential remedy for various skin ailments. That EISO’s therapeutic benefits might be due, in part, to its anti-inflammatory properties are indicated by two recent studies. A clinical study reported that ESIO treatment was tolerated well, and reduced lesion counts, in 90% of acne patients (Moy et al., 2012). This treatment was considered to have efficacy for relieving acne severity using the Global Aesthetic Improvement Scale and Evaluator’s Global Severity Score ratings metrics. The treatment regimen was well tolerated and notable reductions in lesion counts were observed in patients with more severe or inflamed lesions. Another demonstrated that EISO, and its primary constituents, α- and β-santalols can suppress LPS-mediated pro-inflammatory responses and documented its impact on cyclooxygenase activity and cytokine/chemokine expression in skin models (Sharma et al., 2014). We hypothesized that the ability of EISO and its santalol constituents to suppress dermal proinflammatory cytokine production would be of use to treat various inflammation and autoimmune-mediated skin conditions, including psoriasis.

In this report, we evaluated the ability of a well-characterized EISO to relieve psoriasis symptoms in a small cohort, to revert psoriatic characteristics of reconstituted organotypic psoriasis skin models, and to antagonize production of pro-inflammatory cytokines and chemokines and markers of proliferation by comparing responses of parallel normal (non-psoriatic) skin models.

## Materials and Methods

### Phase 2 Clinical Trial

A single-center, open-label safety, tolerability and efficacy trial of an anhydrous serum formulation of EISO at one dose level (10% w/w EISO in a caprylic/capric triglyceride, dimethyl isosorbide, ethoxydiglycol formulation) for the treatment of mild to moderate plaque psoriasis in adult subjects was conducted at Fremantle Dermatology in Australia. The study was carried out with the recommendations of the Bellberry human research ethics committee (HREC) in accordance with the national health and medical research council’s national statement on ethical conduct in human research (Application No: 2015-12-873) and the trial was registered with the Australian therapeutic goods administration (Application ID: CT-2016-CTN-01885-1 v3). All subjects gave written informed consent in accordance with the Declaration of Helsinki. The study enrolled 12 subjects over 18 years old, each with a maximum of 10% body surface area affected by plaque psoriasis. The study medication was applied twice a day for up to 28 days. The Investigator Global Assessment (IGA; Langley et al., 2015) was used to evaluate severity of disease and response to treatment during the 28-day treatment period.

### EISO formulation

East Indian Sandalwood oil (lot: PISO-110904SD/SA), an essential oil obtained by distillation from the heartwood of plantation-grown *S. album* trees, was obtained from Santalis Pharmaceuticals (San Antonio, TX, USA). According to the Certificate of Analysis, the concentrations of α- and β-santalol were 49.0 and 20.8%, respectively, and were in compliance with the International standard for Indian sandalwood oil (ISO 3518:2002). The principal components of EISO are shown in **Table 1**. EISO was diluted in dimethyl sulfoxide (DMSO) as a vehicle, and the final concentration of DMSO used in the experiments (1:100,000) had no adverse effects on the cells.

**Table 1:** Chemical composition of East Indian sandalwood oil (EISO)^a^.

Sesquiterpenes	EISO (%)^b^
(Z)-α-Santalol	41–55
(Z)-β-Santalol	16–24
(Z)-Nuciferol	0.8–3.0
epi-β-Santalol	3.5–4.1
(Z)-α-trans-Bergamotol	5.0–6.7
(Z)-β-curcumen-12-ol	0.5–1.9
β-Santalal	1.0–2.4
(Z)-lanceol	1.4–5.6
(E)-β-Santalol	3.5–4.1
β-Santalene	0.7–1.5

*^a^EISO component analysis was performed by Australian Sandalwood Oil Company, Albany, WA, USA.*

*^b^% Average concentration of sesquiterpenes in EISO, mass per volume.*

### Human Full-Thickness Skin Model

Reconstituted full-thickness normal human skin and psoriatic phenotype (MatTek Corp., Ashland, MA, USA) were incubated in the manufacturer’s assay media (Grossman et al., 1989; Nograles et al., 2008) supplemented with or without EISO 0.001–0.002%. Media, with or without supplementation, were changed every second day. At the specified time points, specimens were collected for histologic analysis using H&E and immunohistochemistry (IHC), and the culture media was collected for cytokine/chemokine enzyme-linked immunosorbent assay (ELISA) analysis.

### Antibodies and Reagents

Monoclonal antibodies (mAb) for Ki67 (8D5) and psoriasin (47C1068) were purchased from Cell Signaling Tech (Danvers, MA 01923) and Abcam (Cambridge, UK), respectively. The non-steroidal anti-inflammatory (NSAID), ibuprofen (IB), was obtained from Sigma–Aldrich (St. Louis, MO, USA). IHC staining of reconstituted psoriatic and normal human tissue models was conducted. Deparaffinized sections were subjected to antigen retrieval, followed by incubation with primary antibodies (1:500 dilution for Ki67 mAb and 1:200 dilution for anti-psoriasin mAb). Bound antibodies were detected by DAB staining using Ventana universal secondary antibody (Ventana Medical System, Tuscan, Arizona). All stained slides were digitally imaged at magnification equivalent to ×20. Representative fields were scored by an independent pathologist.

### ELISAs

Sandwich ELISAs for CXCL5 (ENA-78), GM-CSF, IL-1β, IL-6, IL-8, and MCP-1 (RayBiotech, Norcross, GA, USA) were performed on culture media from the reconstituted normal and psoriatic tissue models (with and without treatment with EISO) according to manufacturer’s instructions. Standard curves were constructed with supplied standards to allow conversion of OD_450_ nm absorbance readings of experimental samples to pg/ml of the respective factors. All samples were assayed in triplicate.

### Statistical Analysis

Results were compared using a one-way analysis of variance (ANOVA) followed by a Bonferroni post-test comparing only the pairs of interest if ANOVA *p*-values were significant. Enzyme-linked immunosorbent assays: the post-test results are shown as ^∗^*p* < 0.05, ^†^*p* < 0.01, ^‡^*p* < 0.001.

## Results

### Phase 2 Clinical Study

**FIGURE 1:**
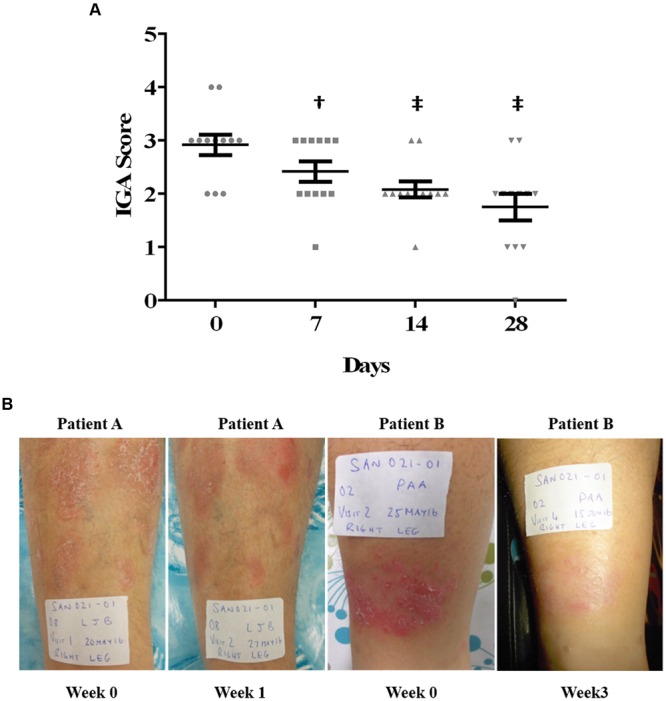
**East Indian Sandalwood Oil (EISO) improves psoriasis lesion severity. (A)** Response of psoriasis lesions from 11 patients to EISO treatment. Scatter plot shows each patients’ lesion IGA score at start of study (circles, Day 0) and at weekly intervals (squares, Day 7; triangles, Day 14; upside down triangles, Day 28). The mean score (black horizontal line) ±SEM is shown for each time point. ^†^*p* < 0.01; ^‡^*p* < 0.001 vs. Day 0 initial scores. **(B)** Representative images from two patient lesions treated with EISO exhibiting a reduction in the severity of psoriatic plaques after either one (Patient A) or three (patient B) weeks of treatment.

In nine of the eleven evaluable subjects in the trial, the severity of psoriatic plaques was reduced by end of study (**Figure 1A**). One patient withdrew from the study with a mild adverse event after 3 weeks on study. The mild skin reaction at the application site resolved upon withdrawal of the study medication. The average IGA score of the cohort was significantly reduced by 1 week on study and continued to improve at 2 and 4 weeks on study. Overall, 64% of the subjects (7/11) demonstrated a ≥1.0 reduction in their IGA score during the 28 day treatment period. Two examples of lesions prior to and after either one (Patient A) or three (Patient B) weeks of treatment scored as markedly improved are presented (**Figure 1B**). Two additional patients demonstrated moderate improvement of their psoriatic plaque. These clinical observations indicated that EISO can provide symptom relief for psoriasis sufferers, prompting us to explore the mechanisms underlying EISO’s ability to mediate these responses.

### EISO Treatment of Normal and Psoriatic Reconstituted Human Skin

**FIGURE 2:**
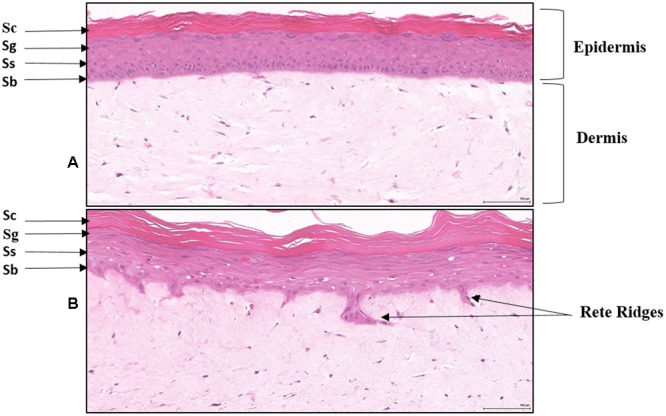
**Histology of Reconstituted normal epidermal tissue vs reconstituted psoriatic tissue: H&E stained cross-sections showing reconstituted skin mimics normal epidermis organization and differentiation histology **(A)**.** The tissue morphology of the psoriatic tissue model closely parallels that of human psoriatic skin **(B)**. The epidermis contains hyper proliferative basal keratinocytes stratum basale (Sb) with regular elongation of the rete ridges (psoriatic epidermal hyperplasia), and spinous (Ss), granular (Sg), and stratum corneum (Sc) layers. The dermis contains psoriatic fibroblasts (20X). Scale bar = 100 μm.

To assess whether the clinical observations above could be recapitulated in an *in vitro* skin assay, reconstituted organotypic psoriatic and normal skin preparations were treated in the absence or presence of EISO. Reconstituted skin samples were harvested at 2, 4, and 8 days after treatment to assess the impact of EISO on normal skin or psoriatic phenotype. Histologic analysis of H&E-stained sections were used to define epidermis and keratin layer morphology (**Figure 2**). The epidermis of normal skin specimens exhibited well-differentiated characteristics of a defined stratum basale layer under a stratum spinosum and stratum granulosum layer topped by a discrete stratum corneum layer (**Figure 2A**). The epidermis of the psoriatic skin specimens showed disease characteristics including defined Rete ridges, a multicellular thick stratum basale layer, poorly defined stratum spinosum and stratum granulosum layers, and a thickened stratum corneum layer (**Figure 2B**). These observations ae consistent with previous reports indicating that the MatTek reconstitute skin specimens accurately model normal and psoriatic epidermal features (Grossman et al., 1989; Nograles et al., 2008).

**FIGURE 3:**
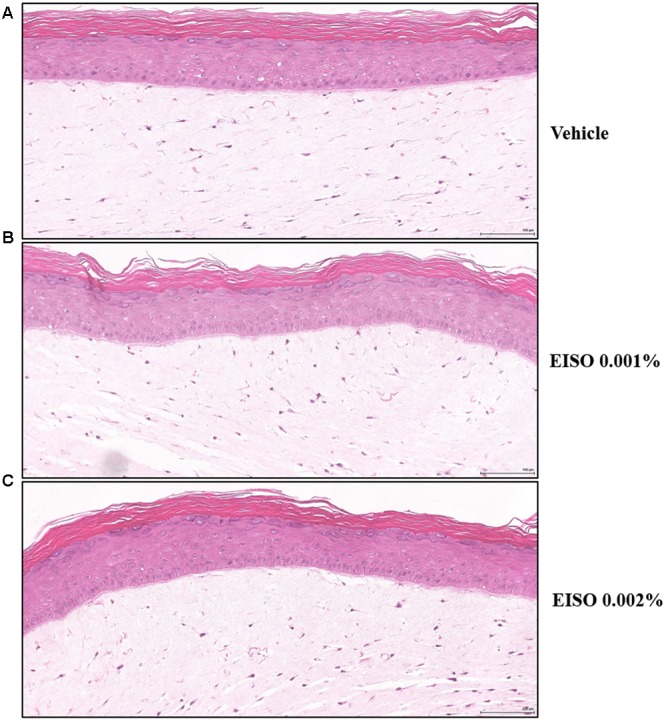
**Normal Epidermal tissue is not affected by EISO treatment.** Reconstituted normal skin model, vehicle treated **(A)**, or treated with EISO at 0.001% **(B)** or 0.002% **(C)** for 4 days. Scale bar = 100 μm.

**FIGURE 4:**
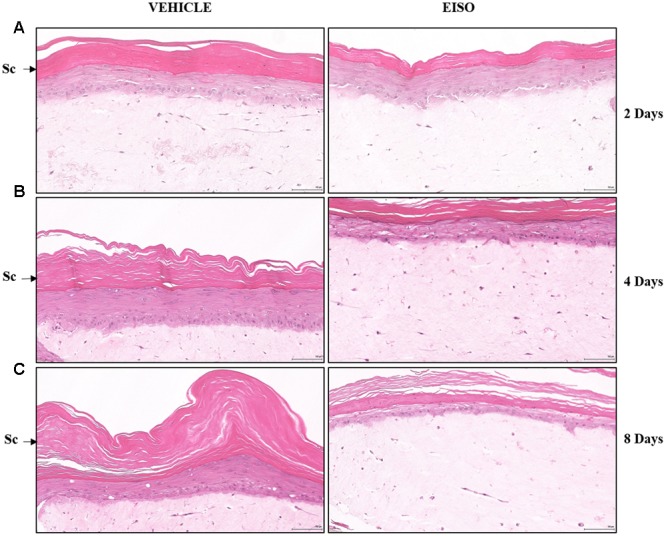
**East Indian Sandalwood Oil treatment restores epidermal architecture of reconstituted psoriatic tissue.** Psoriatic tissue vehicle treated (left images) or treated with EISO (Right images) at 0.002% for 2 days **(A)**, 4 days **(B)** or 8 days **(C)**. Representative images of decreased epidermal layer thickness (Sc) and better organized stratum basale (Sb) in the EISO-treated samples. Scale bar = 100 μm.

Simple observation of H&E-stained samples suggests that EISO was able to revert psoriatic pathology. Reconstituted normal epidermal tissue model showed no change in the epidermal layers after treatment with EISO 0.001–0.002% for 4 days (**Figure 3**). When EISO treated, the reconstituted psoriatic tissue exhibited a normalized epidermal architecture (**Figure 4**). Epidermal thickness was decreased after just 2 days of treatment and stratification of the epidermal layers appears to better recapitulate structures seen in normal skin model.

**FIGURE 5:**
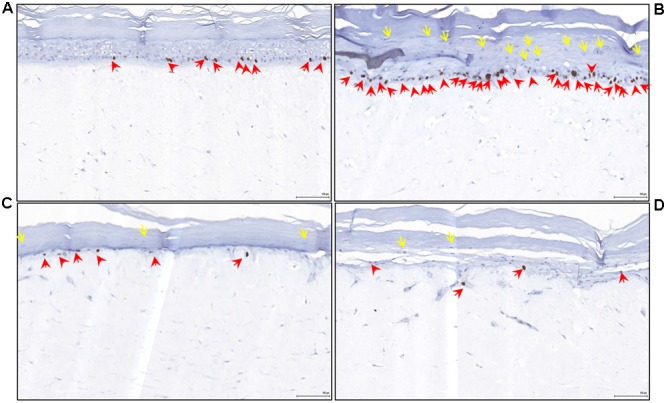
**East Indian Sandalwood Oil treatment (4 days) reverses epidermal inflammation and hyperproliferation in psoriatic skin model.** Reconstituted normal and psoriatic skin samples were cultured in media ±EISO. Immunohistochemical staining of reconstituted psoriasis tissue model showing Ki67 as marker of proliferation restricted to the stratum basale layer (red arrows). **(A)** Reconstituted normal skin 3D cultures, **(B)** untreated psoriatic skin, **(C)** psoriatic skin +0.001% EISO, **(D)** psoriatic skin + 0.002% EISO after 4 days. In addition, the increased keratinocyte nuclei observed in stratum corneum of psoriatic epidermis (yellow arrows) is decreased in EISO treated psoriatic skin cultures. These nuclei are indicative of impaired terminal differentiation of keratinocytes. Scale bar = 100 μm.

Psoriasis is characterized by a hyper-proliferative stratum basale, and using Ki67 as a marker of proliferative cells, we observed a twofold increase in the number of proliferative cells in the stratum basale of the reconstituted psoriatic tissue models (**Figure 5B** and **Table 2**). The proliferative index of the EISO-treated psoriatic skin model samples was suppressed to levels below that observed in the normal skin model sample. The number of Rete ridges correlated with Ki67-positive nuclei total count in psoriatic tissue vs control epidermal tissue (**Figure 5B** vs. **Figure 5A** and **Table 2**). In addition, the increased keratinocyte nuclei observed in stratum corneum of psoriatic epidermis (yellow arrows), indicative of impaired terminal differentiation of keratinocytes, is decreased in EISO treated psoriatic skin cultures (**Figure 5C**: EISO 0.001% and **Figure 5D**: EISO 0.002%).

**Table 2:** Ki67 average count and Rete ridges count in Psoriasis (PS) and Epidermal (EPI) control tissue models treated with ±EISO at 0.002% for 4 and 8 days (4D and 8D) for IHC analysis.

ID	Ki-67 Avg count ± SEM	Rete Ridges
4D-EPI-C	11.25 ± 2.60	0
4D-PS-C	28.75 ± 2.06	21
4D-PS-E 0.002%	1.5 ± 0.57	2
8D-EPI-C	16.00 ± 4.70	1
8D-PS-C	32.75 ± 10.42	27
8D-PS-E 0.002%	1.25 ± 0.62	1

**FIGURE 6:**
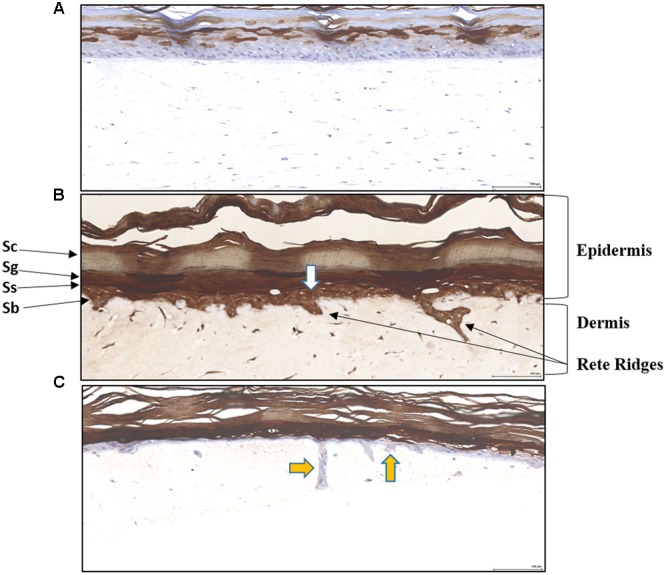
**East Indian Sandalwood Oil treatment results in expression level and cellular distribution pattern of psoriasin.** Vehicle-treated reconstituted normal epidermal tissue model **(A)**, vehicle-treated **(B)**, and EISO-treated reconstituted psoriatic skin model **(C)**. Psoriasin is localized in hyperplastic epidermis and reduced after 8 days of EISO treatment at 0.002% (yellow arrows). Scale bar = 100 μm

The ability of EISO to revert psoriatic pathology is further documented by immunohistochemical analysis of the cellular distribution and patterns of expression of the antimicrobial S100-family member, psoriasin (S100A7) within reconstituted normal (**Figure 6A**) and psoriatic specimens (**Figures 6B,C**). Psoriasin is known to be localized in hyperplastic epidermis of skin in the cytoplasm of keratinocytes (Madsen et al., 1991; Morizane and Gallo, 2012). When the reconstituted psoriatic tissue was treated with 0.002% EISO for 8 days, psoriasin levels were reduced (**Figure 6**).

### Anti-inflammatory Properties of EISO on Reconstituted Psoriasis Tissue

**FIGURE 7:**
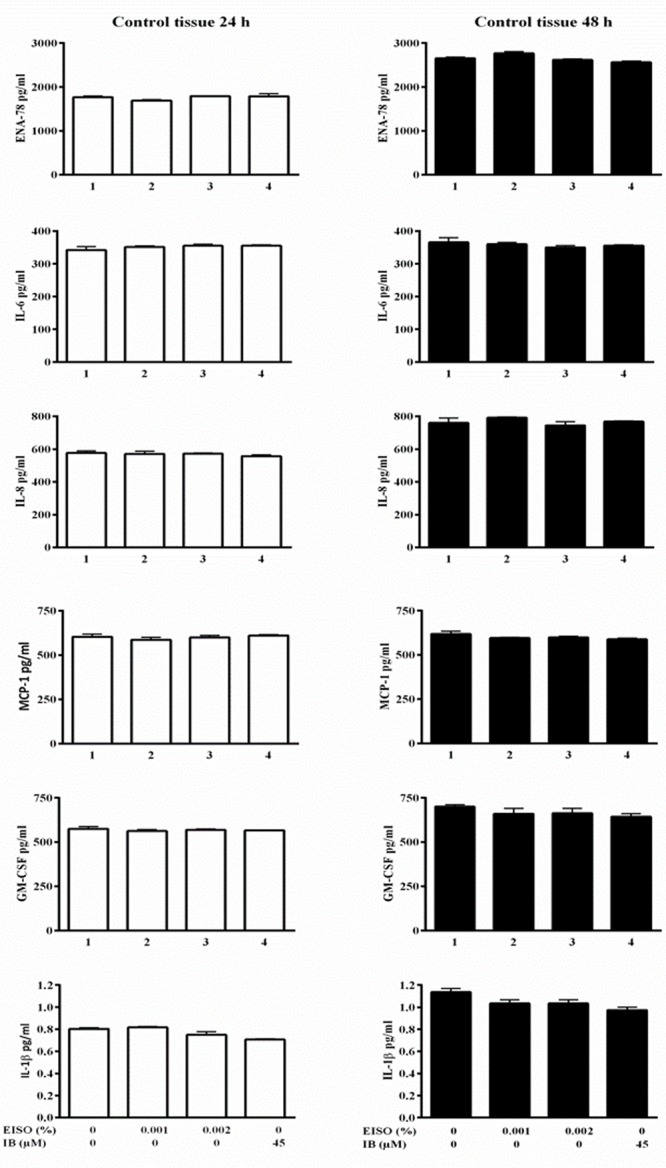
**Cytokine/chemokine levels from reconstituted normal epidermal model is basally low and not affected by EISO or IB treatment.** Reconstituted normal epidermal tissue was treated ±EISO and IB at the indicated concentrations for 24 h (left) or 48 h (right). Accumulation of ENA-78, IL-6, IL-8, MCP-1, GM-CSF, and IL-1β were determined from conditioned media by enzyme-linked immunosorbent assay, expressed as pg/ml.

**FIGURE 8:**
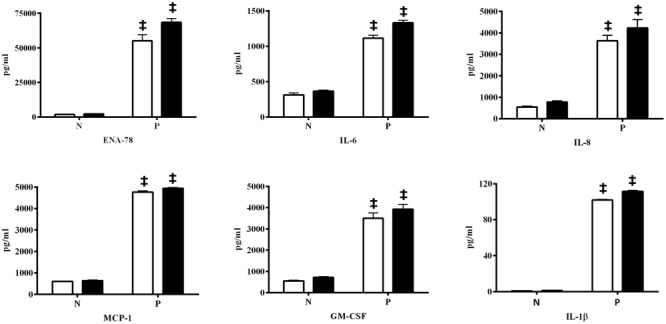
**Skin model of psoriasis exhibits increased potential key pro-inflammatory cytokines/chemokines.** Accumulation of ENA-78, IL-6, IL-8, MCP-1, GM-CSF, and IL-1β were determined from conditioned media by ELISA from reconstituted normal epidermal tissue (N) and reconstituted psoriasis tissue (P) after EISO treatment for 24 h (open) and 48 h (filled), expressed as pg/ml, ^‡^*p* < 0.001 vs. Normal epidermal tissue (N).

**FIGURE 9:**
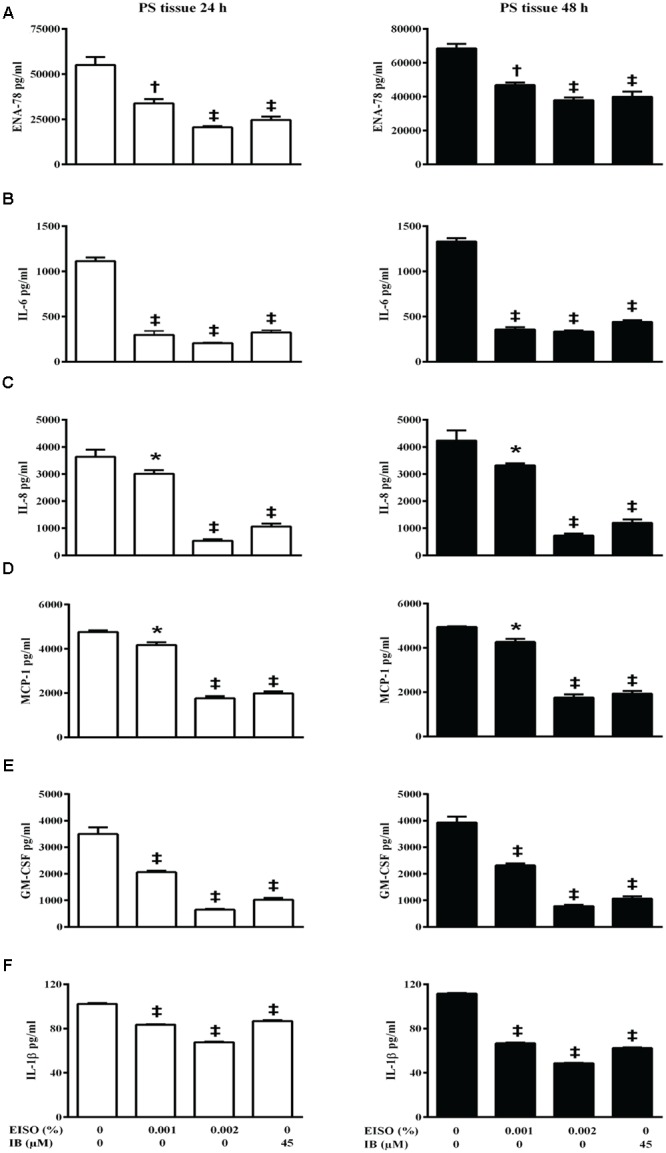
**East Indian Sandalwood Oil suppresses pro-inflammatory cytokines/chemokines production in reconstituted psoriasis skin model.** Reconstituted psoriasis skin models were treated ±EISO or ibuprofen (IB) at the indicated concentrations for 24 h (left) and 48 h (right). Accumulation of ENA-78 **(A)**, IL-6 **(B)**, IL-8 **(C)**, MCP-1 **(D)**, GM-CSF **(E)**, and IL-1β **(F)** was determined from conditioned media by ELISA. Cytokines/chemokines accumulation from psoriasis tissue treated with EISO and IB were compared with the levels of respective Cytokine/chemokine collected from untreated psoriasis tissue, expressed as pg/ml, ^∗^*p* < 0.05, ^†^*p* < 0.01, ^‡^*p* < 0.001 vs. Reconstituted psoriasis skin.

Psoriatic lesions are driven by intrinsic pro-inflammatory reactions. An examination of pro-inflammatory cytokine/chemokine production measured in conditioned media from control reconstituted skin models demonstrated that the normal skin samples produce very low levels of the indicator factors, and were not affected by EISO or ibuprofen treatment (**Figure 7**), while the psoriatic skin models produce significantly higher levels of all six tested factors after 24 and 48 h in culture (**Figure 8**). Of these, IL-1β level was increased >100-fold. Expression of ENA-78 was detected at the highest concentration (69 ng/ml); a >30-fold increase. MCP-1, IL-8 and GM-CSF levels were elevated ≥ 5-fold, and IL-6 level was increased > 3-fold. Treatment of the psoriatic tissue model with EISO exhibited equivalent dose dependent suppression of 6 tested cytokines/chemokines, in some cases to levels indistinguishable from that seen in the normal skin model samples (**Figure 9**). At 0.001% EISO suppressed ENA-78 by 31%, IL-6 by 73%, IL-8 by 21%, and MCP-1 by 13.6%. GM-CSF and IL-1β levels were decreased by ≥ 40%. At 0.002% EISO suppressed ENA-78 by 45%, IL-6 by 75%, IL-8 by 83%, MCP-1 by 65%, GM-CSF by 80%, and IL-1β by 56%. The robust suppression of pro-inflammatory cytokine production suggests that the ability of EISO to revert the psoriatic phenotype might be due to the previously reported ability of EISO to effectively suppress epidermal inflammatory cytokine production (Sharma et al., 2014).

## Discussion

Psoriatic lesions are areas of severe inflammation and erythema, with abnormal differentiation and hyperproliferation of the keratinocytes that is caused by a pathogenic immune response driven by reciprocal cytokine, chemokine and growth factor interactions between cells of the epidermis and the innate and adaptive immune system (Lowes et al., 2007). A variety of topical and systemic anti-inflammatory agents, including phytotherapeutics alone or in combination are used to manage psoriasis (Rahman et al., 2012). In spite of a range of options, effective treatment of psoriasis can be challenging. While short-term treatments provide temporary symptom relief, over time use of these conventional treatments can cause debilitating side effects such as immunosuppression, cutaneous atrophy, organ toxicity and can be carcinogenic (Yuqi, 2005; Traub and Marshall, 2007). While, more recently developed biological agents offer improved anti-psoriatic therapeutic responses, they too run the risk of having adverse side effects, are expensive, and might result in development of resistance or tolerance (Gottlieb et al., 2008).

Natural products may provide effective alternatives for psoriasis treatment while offering less severe side effect profiles. Unlike the high target selectivity and activity of current pharmaceuticals used to treat psoriasis, herbal-derived agents offer a litany of novel bioactive molecules and the potential of using several interactive mechanisms of action that are not found in targeted synthetic agents (Lebwohl et al., 2005; Weger, 2010; Deng et al., 2013). Lack of regulatory approval and patient adoption of NPs for psoriasis treatment is due to the limited information regarding their efficacy and safety (Herman and Herman, 2016). Clearly promotion of herbal products as psoriasis treatments must be supported by rigorous assessment of mechanisms of action, and reliable clinical trials using standardized therapeutic formulations.

East Indian Sandalwood oil has been used since antiquity for inflammatory and eruptive skin disorders, but what we know of its anti-inflammatory properties are anecdotal, and limited by lack of uniform standardization and characterization of most preparations. The sesquiterpene alcohols (*Z*)-α-santalol and (*Z*)-β-santalol constitute approximately 70% of EISO (Christenson et al., 1981). International standard (ISO 3518:2002(E)) for East Indian Sandalwood oil specifies 41–55% (*Z*)-α-santalol and 16–24% (*Z*)-β-santalol. Both santalol isoforms are biologically active, and are being explored for possible chemo-preventative, and anti-viral activites (Kaur et al., 2005; Ochi et al., 2005; Dwivedi et al., 2006; Kim et al., 2006). Although EISO’s mechanisms of action are not completely elucidated, santalols derived from EISO have been shown to have anti-proliferative properties (Lee et al., 2015) as well as significant anti-inflammatory properties in skin models that are linked to suppression of prostaglandin and thromboxane production and cytokine/chemokine expression (Sharma et al., 2014). We hypothesized that the ability of EISO to suppress pro-inflammatory events by keratinocytes and dermal fibroblasts, as well as its antiproliferative properties, would make EISO useful as a potential treatment of a variety of pathophysiologic inflammatory and autoimmune skin ailments, including psoriasis.

Consistent with the afore described properties, the interim results reported here from a small, on-going proof-of-concept Phase 2 clinical trial in patients with mild to moderate psoriasis indicate that a 10% EISO serum formulation administered topically twice a day for 28 days, is well-tolerated and helpful in reducing disease severity, as measured by improvement in the IGA scores (Langley et al., 2015). This proof-of-concept study was designed to assess efficacy/safety of EISO treatment of psoriasis lesions. The promising results seen in this test cohort are being validated in two, 60 patients randomized, placebo-controlled trials to be conducted in parallel in the US and Australia. This human trial is being continued, and additional, larger, placebo–controlled trials are being planned to further determine the clinical potential for an EISO-based treatment of psoriasis. These clinical results are supported by *in vitro* studies using the same EISO preparation indicating that the clinical responses are due to the anti-inflammatory properties of EISO.

Using reconstituted normal and psoriatic skin organoid cultures, we observed improved histologic pathology with improved epidermal stratification, and reduction in number of and size of Rete ridges, upon EISO treatment. Psoriasis is characterized by a hyper-proliferative stratum basale, and using Ki67 as a marker of proliferative cells, we observed down-regulation of the proliferative index of the EISO-treated psoriatic skin model samples, to levels below that observed in the normal skin model. This intriguing observation is consistent with our previous report that santalols can interact with, and disrupt microtubules, and so, in addition to acting as an anti-inflammatory agent, may act to directly suppress cell division (Lee et al., 2015).

Psoriasin is highly expressed in psoriatic lesions (Madsen et al., 1991; Morizane and Gallo, 2012), and other hyper proliferative and inflammatory disorders (Algermissen et al., 1996). Elevated expression of psoriasin in psoriatic plaques (Morizane and Gallo, 2012), as well as in other hyper-angiogenic conditions, has validated it as a marker to assess in the context of psoriasis (Vegfors et al., 2016). Additionally, psoriasin is a chemotactic for CD4+ T cells and neutrophils (Jinquan et al., 1996), and is induced by psoriasis-associated inflammatory cytokines that include IL-1β, IL-17, IL-22, and TNF-α (Liang et al., 2006). Our demonstration that EISO treatment reverted psoriatic pathology by immunohistochemical analysis of the cellular distribution and expression level of psoriasin in reconstituted organotypic psoriatic tissue model is again consistent with the anti-inflammatory properties attributed to EISO.

An examination of a panel of psoriasis indicator pro-inflammatory cytokine levels in the reconstituted skin models demonstrated very low levels in the normal skin model and profoundly higher levels in the psoriatic skin model, and that EISO treatment suppressed cytokine/chemokine production, in some cases to levels indistinguishable from that seen in the normal skin model samples. While these results are indicators that EISO can suppress intrinsic inflammatory processes in psoriatic dermis and epidermis, one of the primary drivers of psoriasis pathogenesis are a class of T helper cells called Th17 cells (Boniface et al., 2007; Wilson et al., 2007; Lowes et al., 2008; Glaser et al., 2009; Kagami et al., 2010). These IL-17-producing leukocytes are thought to initiate the run-away cycle of IL-1β, IL-6, IL-8, and TNF-α production by the skin cells that leads to hyperplasia, elevated psoriasin production, and dysregulted differentiation of the keratinocytes, and further recruitment of proinflammatory immune system components (van Beelen et al., 2007; Nograles et al., 2008; Morizane and Gallo, 2012). This results in recruitment of more IL-17-producing T cells and neutrophils into the skin. We do not report on IL-17 as the reconstituted organotypic normal and psoriatic skin models used the lack immune cell components. Rather, we conclude that the diseased state is imprinted on the psoriatic dermal fibroblasts used to construct the organoids, and that EISO is able to suppress constitutive expression of these downstream cytokines and chemokines drive the psoriatic phenotype. These preliminary clinical and *in vitro* findings strongly support follow-up studies to expand the duration of the treatments and to directly document expression of molecular markers indicative of restoration of a normal epidermal architecture. Subsequent studies with these models will facilitate further mechanistic understanding of how psoriatic dermal fibroblasts are able to induce hyper-proliferation of epidermal cells derived from normal skin.

## Conclusion

Consistent with the afore described properties, the interim results from a small, on-going proof-of-concept Phase 2 clinical trial in patients with mild to moderate psoriasis indicate that a EISO administered topically, is well-tolerated in reducing disease severity. EISO treatment of psoriatic reconstituted human skin, improved histologic pathology with improved epidermal stratification, reduction in number of and size of Rete ridges and down-regulation of the proliferative markers like Ki67 and psoriasin to the levels that observed in the normal skin model. A panel of psoriasis indicator pro-inflammatory cytokine levels demonstrated very low levels in the normal skin model and profoundly higher levels in the psoriatic skin model, and that EISO treatment suppressed cytokine/chemokine production, in some cases to levels indistinguishable from that seen in the normal skin model samples. These clinical results are supported by *in vitro* studies using the same EISO preparation indicating that the clinical responses are due to the anti-inflammatory properties of EISO in afflicted lesions. Determining how the various factor(s) produced by the dermal fibroblasts drive the pro-inflammatory response of keratinocytes, what signaling pathway(s) mediate this response, and finally what node(s) of these events are directly affected by EISO will be essential to adoption of EISO-based therapies for treatment of psoriasis.

## Author Contributions

MC, MS, and CL are responsible for study design. MS and MC performed all *in vitro* studies. KG performed the clinical study, Santalis Pharmaceuticals, Inc. (CL, IC, and PC) provided EISO for the experimental use. Manuscript was written by MC, MS, and CL and all other authors provided editorial advice.

## Conflict of Interest Statement

CL, IC and PC are employees of Santalis Pharmaceuticals, Inc. The other authors declare that the research was conducted in the absence of any commercial or financial relationships that could be construed as a potential conflict of interest.
